# Bluetooth 5 performance analysis for inter-vehicular communications

**DOI:** 10.1007/s11276-021-02830-9

**Published:** 2021-11-16

**Authors:** Andrea Aza, David Melendi, Roberto García, Xabiel G. Pañeda, Laura Pozueco, Víctor Corcoba

**Affiliations:** Department for Informatics, University of Oviedo, Gijón, Spain

**Keywords:** Bluetooth 5, Vehicular networks, Ad hoc networks, Communications performance, Internet of Things

## Abstract

Previous work has demonstrated the feasibility of Bluetooth Low Energy (BLE) as an alternative technology for data transfers in inter-vehicular communication (IVC) scenarios. Bluetooth 5.x core specifications enhance the trade-off between energy requirements, communication range and flexibility. In this paper, we aim to analyse the potential of Bluetooth 5 features for VANET applications, proposing a connectionless communication system. By means of field experiments, we evaluate long range and 2 × speed features, defining a set of communication scenarios. This allows us to test both Bluetooth 5.x range and application throughput. The evaluation includes experiments of V2I communications carried out under real highway traffic conditions. The experiments conducted demonstrate that communication ranges up to 300 m may be achieved depending on the communications scenario. The results also show how throughput degrades as the distance between devices increases. The results obtained are used to discuss future work, aimed at deeper analysing Bluetooth 5 features for VANET applications, completing the development of our prototype and evaluating VANET connectionless communications with the features included in the latest Bluetooth 5.2 specification.

## Introduction

In recent years, several systems aimed at enhancing connectivity in Intelligent Transportation Systems (ITS) have been proposed, ranging from dedicated short-range communication standards (DSRC) to Vehicle-to-Infrastructure (V2I) communications via mobile data networks. DSRC technologies have been developed to enable vehicle-to-vehicle (V2V) communications in Vehicular Ad Hoc Networks (VANET). They have been designed for vehicle built-in systems supporting road safety and traffic efficiency applications that do not require high data rate transfers. Bluetooth 4.0 (BLE) has also been proposed for V2V communications in VANETs, as there is still no mass deployment of built-in systems based on DSRC standards. In contrast to Bluetooth classic 2.0 and 3.0 versions, designed to achieve high data rates, BLE was designed as an alternative for low consumption, low data rate and short-range communications.

Considering forecasts indicating that up to 90% of smart-phones were going to include Bluetooth 4.0 technology by 2018, Frank et al. [1] proposed a proof-of-concept application using off-the-self smartphones for V2V communications in 2014. The proposed system was used to demonstrate the feasibility of Bluetooth 4.0 (BLE) as an alternative technology for vehicular communications, taking advantage of the low power consumption, low data rate, Bluetooth 4.0 stack. Frank et al. [1] and Bronzi et al. [2] achieved communication ranges up to 100 m even in city driving scenarios, using Bluetooth connected devices but reporting high delays in data transfers due to the time consumed by the required peering procedure between BLE devices.

Similarly, in this paper, we aim to analyse Bluetooth 5 features in a context of VANET communications, extending previous work and addressing delay issues by means of implementing a connectionless data exchange Bluetooth 5.x based system. There are other technologies different to Bluetooth which may be suitable for VANET communications. For instance, in recent years there has been a great development of LPWAN technologies designed for IoT scenarios which may be used in vehicular communications, such as LoRa [3]. They are particularly interesting because they have been designed for long-range and low-power communications. Nevertheless, they are not generally available nowadays, if they are compared with Bluetooth. Bluetooth is a low-cost, off-the-self technology in modern smartphones and vehicles. This and the new characteristics of the protocol are the reasons why we have adopted Bluetooth. Moreover, recent work only provides partial or unrealistic results of Bluetooth 5.x performance, compared with studies in which BLE 4 was analysed [4, 5].

In this paper we develop a proof-of-concept low-cost system, envisioned to be integrated as a built-in connectivity system for vehicles. Given the fact that Bluetooth is a low-cost technology which is generally available nowadays, the cost and complexity of adopting this technology in vehicular communications is actually moderate. Furthermore, we propose a connection-less communication service because it has many applications in a vehicular environment, including the delivery of information about adverse driving conditions (fog, ice, snow or other adverse weather conditions), traffic density, roadworks, accidents, etc. This information may be broadcasted from traffic information panels or portable emergency beacons and may be forwarded thanks to the usage of Bluetooth mesh communications. As indicated by previous work, smartphones do not support server role implementations, so the results obtained with these devices are actually conditioned by the required pairing process. Thus, in order to implement a connectionless communication system using real devices, we have not used mobile phones. Instead, we have developed a testbed using Nordic Semiconductors nRF52840 development kits that implement the Bluetooth 5.x protocol stack. In order to test both the technology and our system, our experiments were carried out with real devices in large outdoors areas both under static and real motorway driving scenarios.

In the experiments, we measure performance in terms of the received signal strength indicator (RSSI) and the delivery rate. In addition, aiming to test Bluetooth 5.x new features for VANET scenarios, we developed an additional application enabling us to measure the throughput achieved. Inspired by Gomez et al. [6] we analyse how several configuration parameters affect the performance of the protocol, thus identifying the existing relation between configuration parameters and application data rates. Since the idea behind the developed system is to be integrated as a vehicle built-in connectivity system, we have not evaluated the energy consumed for each protocol configuration. In order to collect reliable significant data, all the tests have been conducted under low-medium interference conditions, generated by using a Bluetooth 5.x interfering testbed.

The experiments conducted present promising results showing what can be expected from Bluetooth 5.x for connectionless communications in VANET applications.

The rest of the paper has been organised as follows. In Section 2, we present a review of related work with respect to the evolution of automotive communication capabilities and Bluetooth. In Section 3, we provide a brief BLE technology overview. Section 4 describes the features of Bluetooth 5.x which will be later analysed. In Section 5, we describe the testbed used in the experiments. Section 6 includes the results of our analysis and, finally, in Section 7 we conclude this paper and provide directions for future work.

## Related work

VANET research has accelerated the development of ITS revealing their potential to increase road safety, anticipate potential dangers or improve traffic efficiency.

In [7], Zeadally et al. present an extensive review of VANET research work, including the description of early-stage VANET experiments and deployments, and of emerging VANET simulators. Since then, a lot of work has been done to develop realistic VANET mobility and communication models to face the inherent problems of performing empirical measurements. Zeadally et al. [7] analyse in depth the status of VANET communication technologies at the time, noting the need for standardization promoted by the ITS consortia, and the need for improved, efficient and reliable broadcasting techniques to support VANET communications. The authors propose Bluetooth as a candidate technology for intra-vehicle wireless communications.

Further and recent review work was carried out by Arena and Pau [8]. Their review briefly examines relevant communication systems and technologies that may be used in V2X communications, with the aim of reducing communication delays and extending communication ranges in high mobility, and high-density, vehicular scenarios. V2V communications are expected to be more effective than Original Equipment Manufacturer (OEM) on-board embedded systems [8], pushing the development and dissemination of vehicular communication technologies.

In the evolution of the automotive industry to ITS, automobile technology has incorporated wireless technologies. As Wi-Fi based V2X communication approaches show power consumption, packet delivery ratio and latency problems, the need for native vehicular communication technologies has arisen. Thus, vehicle manufacturers have evaluated the capabilities of cars equipped with dedicated technologies, as General Motors did for DSRC provided vehicles [10]. Although they are not generally available nowadays, LPWAN technologies such as LoRa [3] may be suitable as well for vehicular communications, especially in order to overcome energy constraints in some vehicular scenarios in which a small amount of information needs to be transmitted in long ranges. Nevertheless, OEMs have recently embraced BLE to improve in-vehicle management and control applications. Manufacturers, such as Texas Instruments Semiconductors, identify the automotive market as a significant business opportunity [14], considering BLE as an alternative to enable low-power wireless automotive solutions for connected vehicles. Furthermore, many authors also propose Bluetooth as a solution to overcome the limitations of other technologies in vehicular communication scenarios. For instance, Yang et al. [9] propose VANETS as an emerging application domain for Bluetooth technologies. One of the reasons is the general availability of this technology in modern vehicles, which may even help to reduce the cost of large-scale deployments of V2X systems based on Bluetooth.

Wang et al. [16] include a survey of autonomous driving technologies, including perception improvements in autonomous vehicles by means of VANET communication technologies. These technologies improve on-board sensor limitations in order to avoid accidents caused by decisions made by sensors alone in autonomous driving systems. The survey also states the need for Information Centric Networking (ICN) enabling vehicles to share data gathered by on-board sensors. The authors report that Bluetooth has been proposed and tested for intra-vehicular and inter-vehicular communications.

Kundala et al. [17] have already claimed that vehicular networking communications may involve internal (vehicle to sensor) and external V2X communications.

Mirza and Khan [18] propose a low cost communication system between ECUs (Engine Control Unit) and on-board sensors. They show that wireless BLE systems outperform CAN bus current consumption and communication efficiency. Mirza and Khan present a BLE-based Intra Vehicle Wireless Sensor Network (IVWSN) showing high packet delivery ratios and average RSSI levels higher than in off-the-shelf receivers, demonstrating the need for a CAN system replacement.

Gheorghiu et al. presented in [11] an analysis of the messaging requirements for automatic tolling, weigh in motion and/or automatic classification systems. The authors conducted tests using BLE modules placed in noisy Wi-Fi environments and compared the results observed for BLE and ZigBee communications, extending previous work presented in [12]. In [12] the authors presented a preliminary analysis of packet delays suffered for several packet sizes and interferences created over different Wi-Fi channels. Luo et al. [13] also propose a model to analyse and improve the latency caused by neighbour discovery in BLE networks.

The new features of Bluetooth version 5.x allow semiconductor vendors to improve Bluetooth wireless microcontrollers enabling further triangulation using Angle of Departure (AOD) and Angle of Arrival (AOA); and larger communication ranges (with up to 1.6 km range demonstrated outdoors). In addition to the enhancements in the Bluetooth 5.x version interesting for VANETs, Hernandez-Solana et al. propose in [15] a device discovery process that outperforms previous work by reducing the time consumed and, thus, increasing its potential in high-density networks and dense traffic scenarios.

In this paper, we propose a connectionless V2X system as a suitable networking solution for ICNs. Information about traffic density or weather conditions, as proposed by Barba et al. [19], may be advertised to vehicles without the need of establishing a connection. This is also the approach followed by García et al. [20]. The authors carry out several experiments in order to evaluate the performance of new physical features included in Bluetooth 5.x and their potential for I2V and V2I communications. They perform the experiments in an outdoors area but only under static conditions. In addition, their tests were carried out without interferences.

Our proposal is based on a connectionless mechanism for vehicular communications, taking advantage of Bluetooth 5.x new capabilities. We have designed a broadcaster/observer system that would enable vehicles to perform reduced duty cycle, low-latency, reliable data transfers. Our system takes advantage of connectionless communications, avoiding the pairing process and allowing higher data rates in mobility scenarios. We have included the details of the developed applications, that have been tested using Nordic Semiconductors NRFC52840 DK boards. In order to obtain the most realistic results possible, we designed a testbed with an interference mechanism that reduces the availability of transmission channels and produces low to medium interference conditions. We also present the results obtained in several experiments performed with real devices in large outdoors areas both under static and real motorway driving scenarios. In the experiments we have evaluated the performance of Bluetooth 5.x new physical layers, to provide said connectionless service. The results show that our system is able to achieve longer ranges than those obtained in previous work for connection-based systems. To our knowledge, this paper is the first empirical Bluetooth 5.x based proof-of-concept for V2X communications under real driving conditions.

## BLE overview

BLE is a full protocol stack with a structure that remains in the Bluetooth 5.x specification. BLE was designed as an alternative technology for low power, low data rate single-hop communications. In spite of sharing similar protocol stack structures and operating in the same radiofrequency band, BLE is not backwards compatible with Bluetooth classic (BC) versions using a BR, EDR or HS (AMP) physical transport. BLE included a new LE physical transport service, with a PHY layer operating in the 2.4 GHz ISM band, using 40 RF channels indexed from 0 to 39, with 2 MHz cannel spacing. BLE channels 0–36 are used for data transfers between connected devices, whereas channels 37 (2402 MHz), 38 (2426MHZ) and 39 (2480 MHz) are used for advertising. Only one available data channel is used per transmission during a given time interval. This channel is chosen by using an adaptive frequency-hopping algorithm in order to face interferences and signal propagation issues.

The Link Layer (LL) manages the sequence and timing of transmitted and received frames. It differentiates two roles: master (scanning devices) and slave (advertising devices). BLE allows both connection-based and connectionless communications. Connectionless communications are carried out in time intervals known as advertising events. Advertiser devices transmit BLE packets sequentially using the aforementioned advertising channels. Section 5.2 provides more details about Bluetooth advertising. On the other hand, bidirectional communications require the establishment of a connection between peer devices. In connection-based scenarios, the physical channel is temporarily divided into connection events, with a period of time between consecutive connection events defined by the *connInterval* parameter. This parameter has values multiple of 1.25 ms, ranging from 7.5 ms to 4 s. Within a connection event, transmissions use the same data channel. The LL also provides flow and error control. Each over-the-air data packet includes a 24-bit CRC for transmission error detection. In addition, supervision timeouts are established to detect connection losses due to RF interferences or peers shifting out of range. In connection-based scenarios, the LL provides flow control using a stop and wait mechanism to prevent packet losses by acknowledging sequential packet numbers.

The stack also includes a Generic Access Profile layer (GAP). GAP provides an event based API for the application layer to control the BLE stack, establishing the mode of operation and, thus, handling device procedures to initiate, establish and manage communications with other devices. A given BLE controller supports two GAP role combinations: peripheral/central and broadcaster/observer. The latter is optimum for connectionless advertisement-based communications. Any BLE device may support several roles, but only one can be adopted at a given time.

## Bluetooth 5.x new features

Bluetooth 5.x was released with major improvements, including new PHY variants to provide a LE physical transport service. The updated PHY layers are named LE 2 M PHY and LE Coded PHY, supporting 2 × data rate transfers or up to 4 × communication ranges, compared with the previous BLE specification capabilities. Bluetooth 5.x core specifications enhance the trade-off between energy requirements, communication range and flexibility. The new features included in the Bluetooth 5.x core specification, extending version 4.2, are the following:

- 1. LE 2 M PHY
- 2. LE Coded PHY
- 3. LE advertisement extensions
- 4. High Duty Cycle non-connectable advertising
- 5. LE channel selection algorithm #2
- 6. Slot availability masks

In this Section, we describe the Bluetooth 5.x features that are lately analysed in this paper. It must be taken into account that Bluetooth 5.x does not achieve both increased range and speed simultaneously. Depending on the selected PHY layer, an increased range or an improved speed may be obtained, but not both at the same time. Table 1 shows a comparison of the three physical layers defined in the Bluetooth 5.x core specification.

**Table 1:** Summary of physical layers, modulation and coding schemes [23]

PHY	Modulation scheme	Access header	Payload	Data rate
LE 1 M	1 Msym/s	uncoded	uncoded	1 Mb/s
LE 2 M	2 Msym/s	uncoded	uncoded	2 Mb/s
LE Coded	1 Msym/s	S = 8	S = 8	125 Kb/s

### Double speed using the LE 2M PHY

For the LE 2 M PHY, the usage of a higher symbol rate increases the inter-symbol interference, with a frequency deviation of 370 kHz instead of the 185 kHz deviation used by the LE 1 M PHY. Also, the LE 2 M PHY improves power consumption by reducing the radio-on time required to transmit a given amount of data, thus increasing the spectral efficiency and improving coexistence.

### Long range using LE coded PHY

The LE Coded PHY allows the range to be even quadrupled, compared with Bluetooth 4 ranges, without increasing transmission power. The range at which data can be extracted correctly from a received signal is increased by using an encoding system that enforces the resilience to background noise. Bluetooth defines a BER upper bound at which data can be received correctly of 10E-3.

**Fig. 1:**
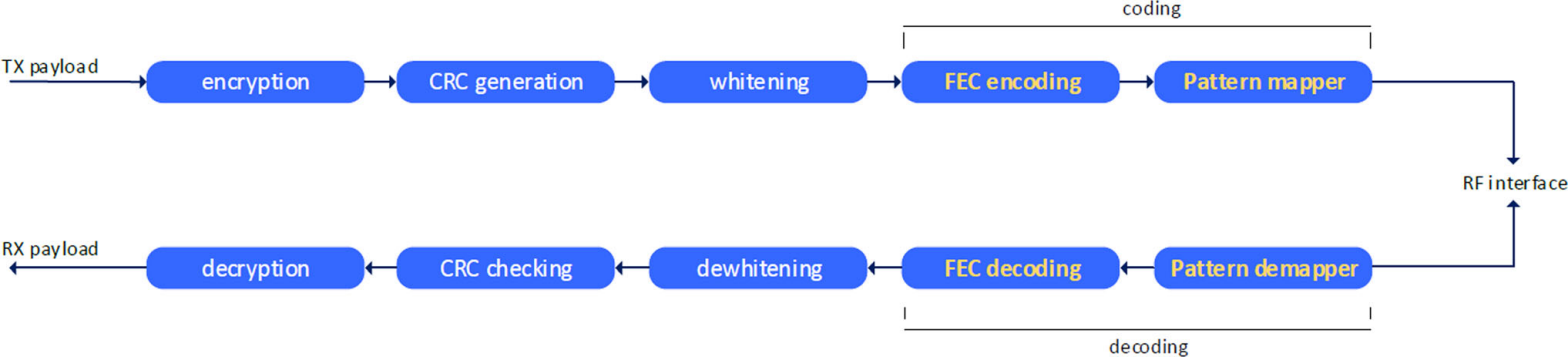
Bluetooth 5.x FEC stream bit processing [21]

The Bluetooth 5.x LE Coded PHY uses Forward Error Correction (FEC), enabling receivers to decode error affected data received at lower SNRs and, thus, extending range. FEC encoding is applied to data streams, adding additional redundant bits to the transmitted packets. Figure 1 shows FEC stream bit processing. The usage of redundancy bits also permits an increase in the sensitivity of receivers, avoiding an increase in transmission power levels to extend range.

The LE Coded PHY may be used with two different coding schemes referred to as S = 2 and S = 8. It is modulated at 1Ms/s, as in BLE, but as raw data rates are reduced down to 500 kbps when using S = 2 encoding and to 125 kbps when using S = 8 encoding, the power consumed is increased due to the required radio-on times. A summary of modulation schemes, coding schemes and raw data rates associated to LE 1 M, LE 2 M and LE Coded physical layers is provided in Table 2.

**Table 2:** Comparison of the three physical layers defined in Bluetooth 5.x core specifications [21]

	LE 1 M	LE Coded S = 2	LE Coded S = 8	LE 2 M
Symbol Rate	1 Ms/s	1 Ms/s	1 Ms/s	2 Ms/s
Data Rate	1 Mbit/s	500 Kb/s	125 Kb/s	2 Mb/s
Error Detection	CRC	CRC	CRC	CRC
Error Correction	NONE	FEC	FEC	NONE
Range multiplier (approx.)	1	2	4	0.8
Bluetooth 5 Requirement	Mandatory	Optional	Optional	Optional

### Bluetooth 5.x extended advertising

**Fig. 2:**
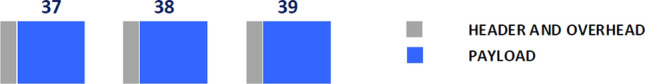
Bluetooth 4 typical advertising sequence over three advertising channels [21]

In Bluetooth 4, advertising packets are 37 bytes long with a header of 6 bytes. BLE defines a transmission of advertising packets in up to three 2 MHz wide advertising channels, numbered 37, 38 and 39. In general, the same payload is transmitted sequentially over the three advertising channels, as shown in Fig. 2.

**Fig. 3:**
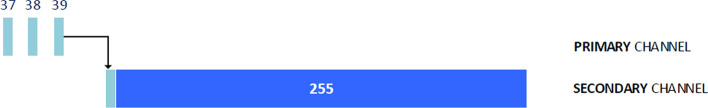
Bluetooth 5 larger advertising packets and channel offloading [21]

The Bluetooth 5.x core specification allows devices to broadcast larger amounts of data than previously, in connectionless scenarios. Packets up to 255 bytes long may be advertised by offloading payloads to one of the 0 to 36 data channels generally used in connection-based scenarios. Only the header is transmitted using BLE 37 to 39 advertising channels (known as primary advertising channels). In this header, the *AuxPtr* field contains a data-channel index and offset information. Receivers use this field to find the offloaded data inserted in auxiliary packets sent over a secondary advertising channel. The way advertising payloads are offloaded to secondary advertising channels is shown in Fig. 3. For use cases requiring broadcasting larger amounts of data, it is possible to chain advertising packets using secondary advertising channels. Offloading advertising data reduces the amount of information transmitted using primary advertising channels, thus reducing contention. In addition, data offloading to secondary channels allows advertising payloads to be transmitted only once. Only the headers containing the *AuxPtr* field need to be sent three times using each primary advertising channel, reducing the amount of data to be transmitted and, thus, the duty cycle.

The Bluetooth 5.x advertising interval has been reduced from 100 ms down to 20 ms for non-connectable advertising, allowing an increase in the rate of over-the-air advertising packets. This is interesting in IVC scenarios, affected by relatively high vehicle speeds and involving shorter temporal transmission windows.

## Evaluation of bluetooth 5 for IVC communications

In this paper, we propose a Bluetooth 5.x broadcaster/observer system that enables vehicles to perform reduced duty cycle, low-latency and more reliable data transfers. Bluetooth 5.x new features present great potential for IVC systems to be built upon the basis of connectionless extended advertisements. Thus, we have designed the system to take advantage of connectionless scenarios, avoiding the time-consuming pairing process and allowing higher data rates in mobility scenarios. This approach also enables our system to achieve longer ranges than those obtained in previous work such as [2] for BLE connection-based systems, as advertising makes our system independent of connection interval timeouts. Our implementation is based on the LE physical transport improvements included in Bluetooth version 5.x.

The results were obtained using an application designed to measure the performance of the protocol in connectionless scenarios, with two boards performing communications in broadcaster/observer modes. Our testbed is based on Nordic Semiconductors NRFC52840 DK boards. These boards support the implementation of Bluetooth 5.x communications in connectionless mode, overcoming the limitations reported in previous work for experiments performed with smartphone-based applications [1]. Our system does not need a connection establishment between peer boards before sending data. As described in Sect. 5.2, we have analysed the range of Bluetooth 5.x communications for connectionless systems using the extended advertisement mechanisms. Furthermore, we have performed an evaluation of the throughput achievable using the Coded PHY and 2 M PHY layers included in Bluetooth 5.x core specifications, as detailed in Sect. 5.3. Finally, in order to obtain realistic results, the testbed used in the experiments includes several devices generating interferences, as described in Sect. 5.4.

The results include measurements performed at different distances between transmitter and receiver boards, both in static and real driving scenarios. They have allowed us to demonstrate the feasibility of Bluetooth 5.x communications under real driving scenarios and, thus, the possibilities of integrating this technology in modern vehicles.

### Potential of bluetooth 5.x for IVC communications

The main advantage of Bluetooth when compared with other protocols is that it is a technology generally available in nowadays smartphones and vehicles. Even though it was traditionally used as a personal area network technology, its potential has notably increased with the new features that have been included in the protocol in the most recent releases. Similarly, Wi-Fi is a technology generally available in smartphones but not in vehicles. Furthermore, previous work has reported problems of this technology in vehicular communication scenarios [8].

If an application designed for a vehicular context would like to be deployed, it could be simply installed in the devices of the users. Similarly, several Bluetooth solutions have been recently adopted in many countries to detect the exposure to individuals who may have COVID-19. In the case of a system designed for vehicular communications, the applications could be very easily installed in smartphones and vehicles having an advanced solution such as Android Auto or Apple CarPlay. The cost of deployment of these solutions would be very low. On the other hand, if a V2I solution would like to be deployed, it would be necessary to install the system in powered road signs or radio beacons. Nevertheless, this is simple in traffic information panels on motorways, as they are already powered and have some type of connection with traffic control centres.

For the aforementioned reasons, we consider that the complexity and cost of deploying a vehicular solution based on Bluetooth are actually moderate.

### Extended advertising range

**Fig. 4:**
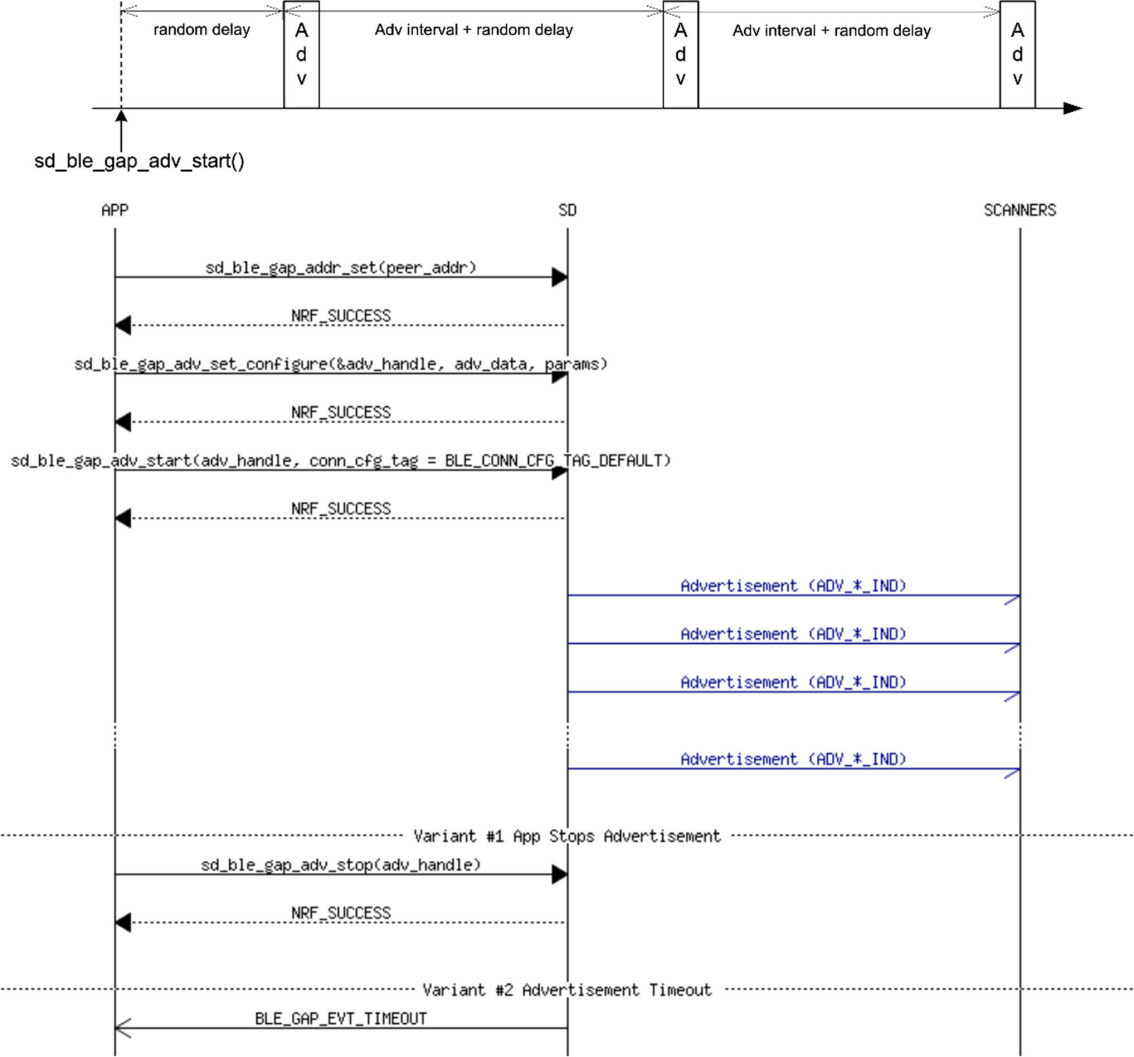
Advertiser timing [25] and advertising message sequence [26]

To evaluate the extended advertising range functionality using connectionless communications, we developed two complementary advertiser and observer applications to run in the aforementioned nRF52840 DK boards, using Bluetooth 5.x ADVB logical transport service. The boards communicate with each other as shown in Fig. 4.

**Fig. 5:**
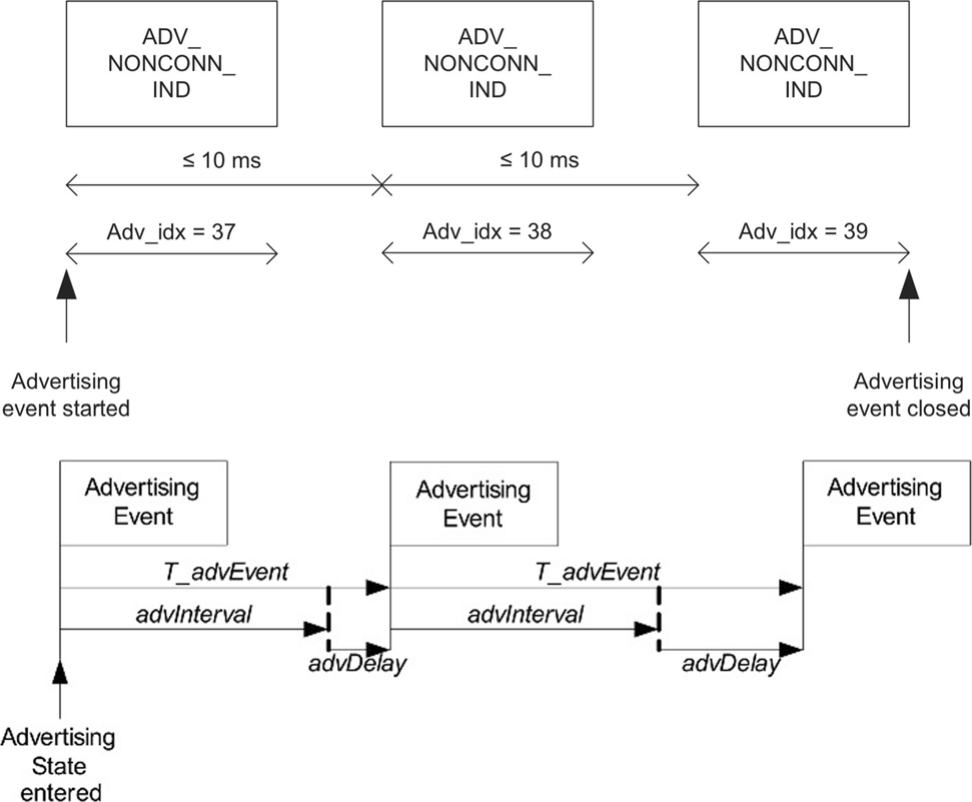
Advertisement flow diagram for the implemented system over primary PHY; non-connectable and non-scannable undirected advertising event [22]

In Bluetooth 5.x, advertising events occur at regular intervals named *advInterval*, modified by a pseudo-random delay ranging from 0 to 10 ms. The *advInterval* is defined by core specifications as an integer value multiple of 0.625 ms in the range between 20 ms and 10,485.759375 s [21]. For the extended advertising IVC system proposed in this paper, we established a medium advertising interval of 187.5 ms, i.e., 300 units of 0.625 ms. Furthermore, advertising events are used by the broadcaster application to send the same 37 bytes packet over each of the available primary advertising channels, as shown in Fig. 5. By the time of this writing, nRF52840 DK boards do not support data offloading to secondary advertising channels [23]. Thus, our system was implemented to advertise 37 bytes packets only using primary PHY channels, which is the size of the payload in standard Bluetooth advertisements.

The observer application was developed to gather RSSI values and their corresponding timestamp for the extended advertisements received. The advertising packets received by the observer are filtered using the expected advertiser address. Thus, we collect log details only for packets coming from the nRF52840 DK advertising board that have been received correctly.

As LE 2 M PHY layer is not permitted by Bluetooth 5.x core specifications for primary advertising, extended advertising range results are presented only for primary advertising using the LE 1 M and LE Coded PHY layers. Also, the nRF52840 DK *softdevice* does not support advertising over LE Coded PHY with a coding scheme S = 2, so the results presented for primary advertising over LE Coded PHY correspond to a coding scheme S = 8.

### Throughput

Bluetooth transmission speeds of 1 Mbps (LE 1 M PHY), 2 Mbps (LE 2 M PHY), 125 kbps (LE Coded PHY+S = 8) and 500 kbps (LE Coded PHY+S = 2) are only theoretical. These performance data rates correspond with radio transmission rates, but they are not the real throughput an application may obtain.

**Fig. 6:**
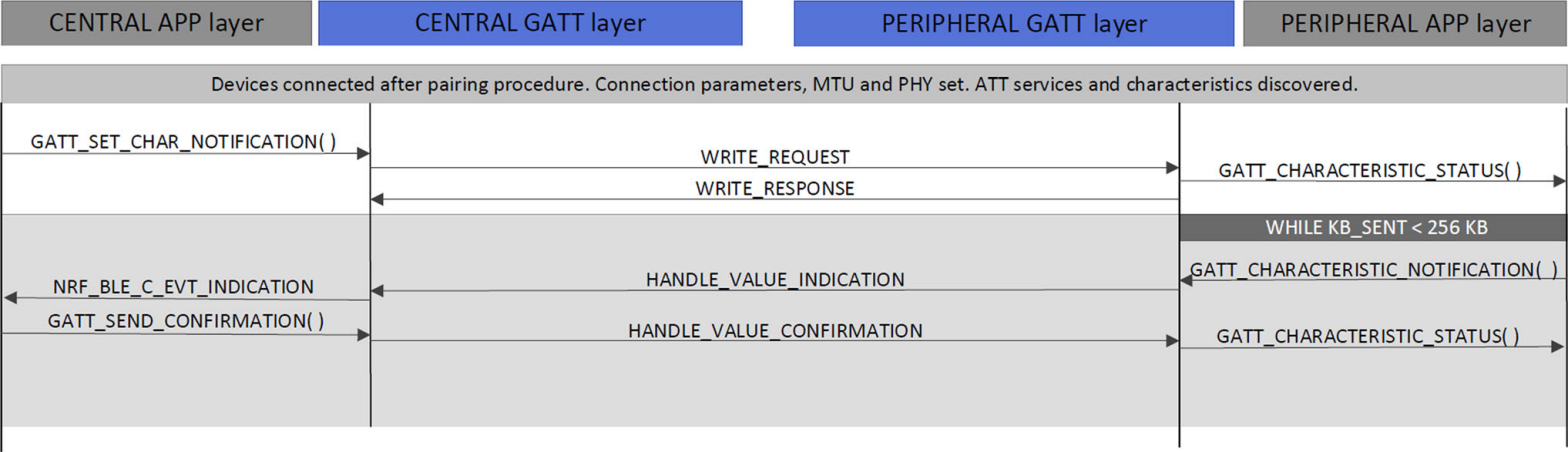
Throughput evaluation application flow diagram, after a connection establishment

It was impossible to evaluate the throughput achievable by connectionless advertising communications over Bluetooth 5.x new PHY layers. LE 2 M PHY is not permitted for primary advertising, and the *softdevice* v6.1 that enables nRF52840 DK boards to perform data offloading to secondary advertising channels, had not been released when the experiments were carried out. In order to face these limitations, we designed a mechanism to measure the throughput achieved with boards communicating in central/peripheral mode using a LE ACL logical transport. Figure 6 shows the design of this system.

In the developed system, one of the boards works in peripheral mode, acting as a server, while the other board works in central mode and acts as a client. Once the central board is instructed to start running the tests, it starts scanning for devices to connect to. On the other hand, the peripheral board starts advertising when it is turned on. This is how both boards connect. Once they are connected, the peripheral board sends notifications periodically. The central board acknowledges these notifications, enabling the peripheral to calculate the number of bytes sent successfully. For the conducted experiments, the connection is terminated once the central board acknowledges a 256 KB data transfer. The board in peripheral mode calculates the throughput once the connection has finished. To perform this calculation, we have only considered the data encapsulated in each over-the-air packet. Once the throughput has been calculated, the peripheral board generates log information including both the calculated value and the time required for the 256 KB data transfer.

**Fig. 7:**
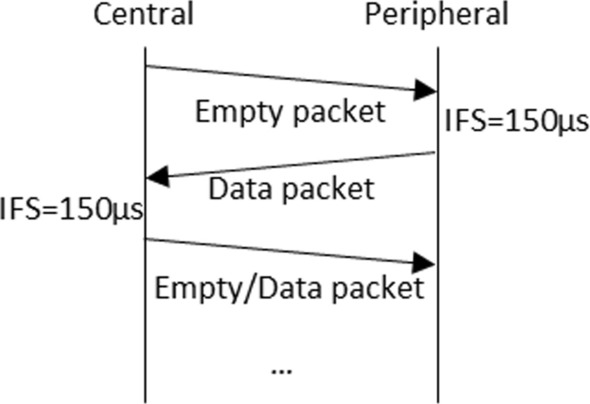
Packet exchange over connection interval

Data packets are sent in connection intervals. BLE defines the connection interval as the time between two consecutive data transfers or connection events, ranging between 7.5 ms and 4 s, as defined by BLE core specifications. Apart from the time required for the central and peripheral boards to send one packet each, the time consumed includes two inter frame space (IFS) periods as shown in Fig. 7. In our implementation we have used the reference value of 150 µs for IFS periods to reduce their effect on throughput. Furthermore, to achieve the highest throughput possible, the Data Length Extensions (DLE) option has been enabled for all the experiments conducted. It is important to consider that, in BLE 4.2, DLE allows peers to continue exchanging packets until the next connection event, thus removing previous limitations on the number of packets per connection interval.

When a packet is lost, the transmitting device waits for the start of the next connection interval to continue sending data after successfully retransmitting a lost packet. Thus, the effective throughput degrades in the presence of errors. For an actual extended advertisement-based system, the throughput achieved would not be affected by the wait-and-stop flow control mechanism or by the time consumed by the central board to acknowledge the packets including the IFS intervals. Thus, the connectionless IVC system proposed in this paper would achieve even higher data rates than those obtained in our experiments. Given the aforementioned limitations, we aim to stablish a minimum reference value for the throughput of the actual extended advertisement based IVC system by using the described central/peripheral communications mode.

### Interference

To benchmark Bluetooth 5.x behaviour under the most realistic operation conditions possible, we designed an interference mechanism to be used in the experiments. As demonstrated in [2], BLE is very resilient to RF interference once a stable configuration has been reached. The improved Bluetooth 5.x channel selection algorithm claims to enhance said resilience. To verify this claim, we developed an interference testbed composed by two advertising Nordic Semiconductors nRF52840 dongles, and a continuous data transfer connection performed by a set of wireless earphones connected to a smartphone. The dongles generated advertisements in the conditions described in Sect. 5.2. They produced 37 bytes advertisements in each primary advertising channel using a medium advertising interval of 187.5 ms. Furthermore, the transmission of data between the smartphone and the earphones allowed us to have a realistic source of interference. We have chosen these elements as a source of interference because they produce exactly the same type of traffic that would be found if the proposed connectionless system is deployed in a real vehicular environment. The traffic of the proposed system would coexist with traffic produced by devices such as mobile phones, car entertainment systems, hands-free appliances or voice gateways. The interferences were always generated close to one of the boards (the observer or the central board, depending on the type of experiment) All these devices allow us to reduce the availability of advertising and data channels, respectively, using realistic usage conditions.

**Fig. 8:**
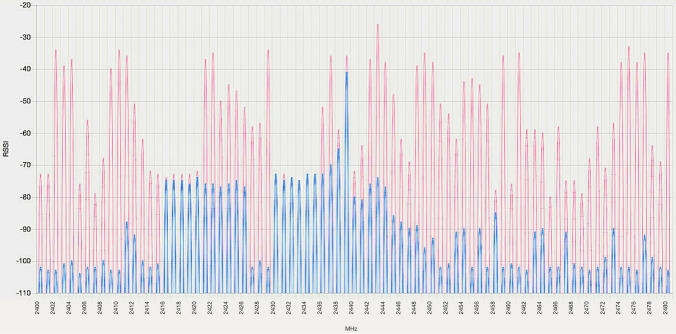
Sample of the interferences generated by the interference testbed. Red line showing maximum RSSI values in the last 5 ms interval and blue line showing instant RSSI values

Figure 8 shows an example of the interferences generated by the described interfering testbed. The red line shows maximum RSSI values observed during the last 5 ms interval and the blue line shows instant RSSI values registered when the sample was taken. These sample values were measured using the Nordic Semiconductors RSSI viewer application.

### Design of the experiments

**Fig. 9:**
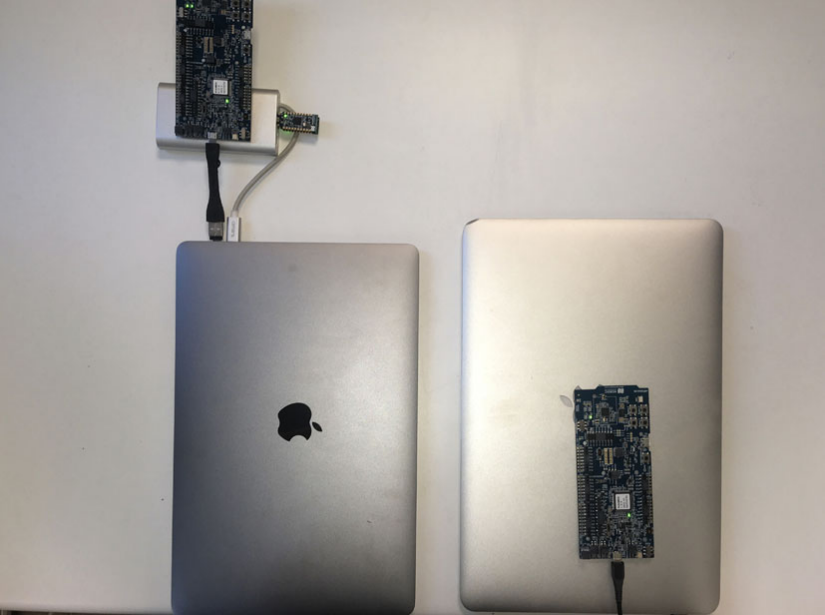
Experimentation testbed

To evaluate both the extended advertising range functionality and the throughput of the system in an IVC context, we conducted several experiments both under static and real driving conditions. Taking into account that our proposal is to have a built-in communications system with integrated antennas, we have performed the experiments outdoors with the testbed boards without any cover. Power consumption of BLE peers in non-connected scenarios varies depending on several parameters, as shown in by Siekkinen et al. [24]. Nevertheless, energy consumption was not considered as a relevant factor because the idea is to have the system installed in vehicles or powered road signs. Thus, both boards were connected to a laptop as shown in Fig. 9, guaranteeing that on-boards antennas delivered the required power levels properly. Figure 9 also shows one of the dongles used to generate interferences, as described in Sect. 5.4 and Table 3 includes a summary of the experiments conducted.

**Table 3:** Summary of the experiments

Feature	General Conditions	PHY	Compared values	Traffic generated	Analysed data
Extended advertising range	Static in large outdoors area with direct line of sight (380 m)	LE 1 M; LE Coded S = 8	Power levels of 0 dBm and 8 dBm; Distance increased in steps of 5 m	37 bytes packets over each primary advertising channel; Medium advertising interval of 187.5 ms; Tests during 5 s; With interference testbed	RSSI levels; Packet loss rates
	Driving in straight segment of major motorway (2 km) with medium to dense traffic; DLE enabled Power level of 8 dBm	LE 1 M; LE Coded S = 8	Vehicle speeds of 80, 90, 100, 110 and 120 km/h	37 bytes packets over each primary advertising channel; Advertising interval of 20 ms (minimum allowed); Non-stop tests; With interference testbed	RSSI levels; Transmission window sizes
Throughput	Static in large outdoors area with direct line of sight (380 m); Power level of 8 dBm	LE 2 M; LE Coded S = 8	MTU sizes of 23B, 158B and 247B; Connection intervals of 7.5, 50 and 400 ms; Distance increased in steps of 5 m	Acknowledged data blocks of fixed size (256 KB); With interference testbed	Time required; Kbps

Regarding the experiments to evaluate extended advertising ranges, we performed tests both under static and real driving conditions. In both types of experiments, we used non-extended advertisements using the nRF52840 DK boards functioning in advertiser/observer modes as described in Sect. 5.2. In addition, the experiments were performed under low to medium interference conditions using the testbed described in Sect. 5.4.

Static experiments to evaluate extended advertising ranges were carried out measuring the performance of the system at fixed positions, increasing the distance between advertiser and observer in steps of 5 m. Measurements were performed at fixed distances because nRF52840 DK boards do not enable us to measure the distance between observer and receiver using GPS positioning, as reported in previous studies [1, 2]. Every time we increased the distance, we initiated a new connectionless communication for at least 5 s. The experiments were conducted in a large outdoors area, enabling us to have a direct line of sight of distances up to 380 m between both boards. Also, data was collected with the advertiser radio configured to transmit using two different power levels, enabling us to analyse how the range achieved is affected by the power level of the transmitter. These power levels are 0 dBm and 8 dBm, which is the maximum output power of nRF52840 DK boards.

**Fig. 10:**
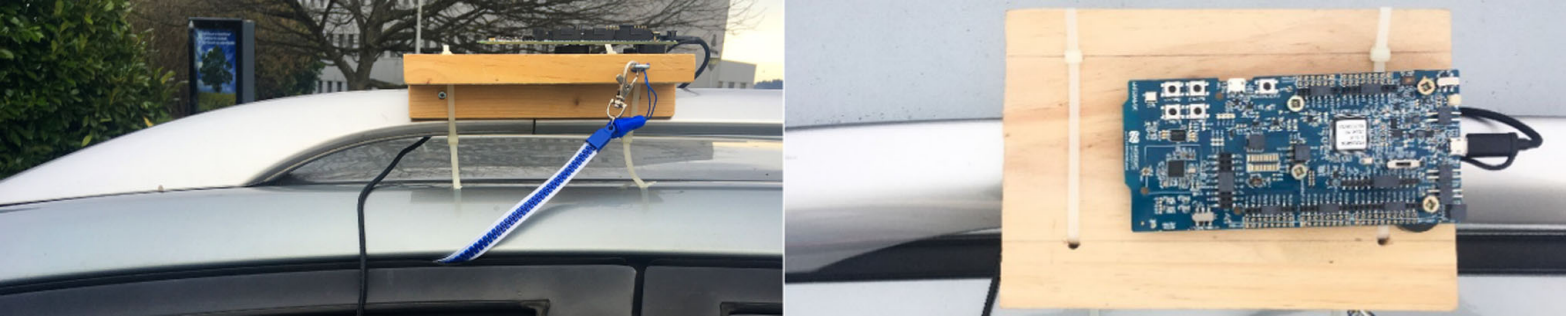
Experimental set up

Also, several experiments were designed to analyse the extended advertising range functionality under real driving conditions. Previous studies [2] performing V2V communication experiments show that the speed at which vehicles are moving does not actually impact the quality of the communications as distance does, provided that the relative speed is kept at 0 km/h. In our case, we performed several driving tests to analyse the effect of varying relative speeds on the communication, for the proposed connectionless system. Using the primary extended advertisement-based system described in Sect. 5.2, we configured the advertiser application to use an advertisement interval of 20 ms, the minimum allowed by core specifications. The advertiser board was attached to a roof rack bar of a car, as shown in Fig. 10. Moreover, the observer board was placed at a fixed position in a bridge over a major motorway, allowing us to measure temporary transmission windows in a V2I communication. We measured RSSI values for each advertisement received from the board attached to the roof of the car every time the car passed under the bridge at different constant speeds: 80, 90, 100, 110 and 120 km/h. To perform the experiments, we chose a straight segment of a major motorway guaranteeing a direct line-of-sight between both boards of at least 2 km. The driving tests were conducted under medium to dense traffic conditions. This includes a constantly changing link budget caused by high volume vehicles occasionally disrupting a direct line-of-sight between both boards. Moreover, the experiments were carried out under real interference conditions, i.e., hands-free devices, vehicles, smartphones and other devices with Bluetooth working or under operating conditions. Also, the interference setup described in Sect. 5.4 was used. In conclusion, the experiments were carried out allowing us to obtain realistic performance results and to check the scalability of the solution.

Several experiments were also conducted to evaluate the throughput using the nRF52840 DK boards communicating in central/peripheral mode, as described in Sect. 5.3. To determine a reference minimum value for the throughput achievable by the proposed connectionless advertisement-based system, we have run a set of tests with different connection configurations. All the tests were static and carried out at fixed distances between the boards, under the same conditions of the static experiments conducted to evaluate the extended advertising range functionality. In order to analyse how distance affects throughput, the boards were gradually separated at steps of 5 m for LE 2 M PHY and of 10 m for LE Coded PHY. Every time we increased the distance, we launched a new set of experiments. The size of connection intervals may be relevant for the throughput of the system. Providing DLE is enabled, larger connection intervals allow devices to transmit data for more time within a given connection interval, thus reducing the number of connection events required to send a fixed amount of data and, also, the overhead and power required to open and close connection events. Nevertheless, large connection intervals decrease throughput in the presence of bit errors, and these errors may occur when the distance between peers is increased. Thus, in the experiments, we analyse the throughput achieved for different connection interval values in order to determine the optimal configuration. We have used connection interval values of 7.5, 50 and 400 ms, as in the Bluetooth Low Energy throughput experiments available in the S140 *softdevice* specification [25]. The attribute protocol (ATT) MTU value is also varied from its minimum of 23B, defined by core specifications, to 247B, corresponding with the maximum over-the-air payload allowed when DLE is enabled. When larger MTU values are used, fewer over-the-air packets are required to send a given amount of data. Thus, both overhead bits and the time required for the transmission are reduced.

## Results and discussion

In order to evaluate the performance of Bluetooth 5.x new features in an IVC context, we used the testbed described in the previous Section and conducted the experiments detailed in Sect. 5.5 both under static and real driving conditions. With the analysis of the data gathered by the boards in different communication scenarios, we have been able to draw a set of conclusions that are presented in this section.

### Extended advertising range under static conditions

**Fig. 11:**
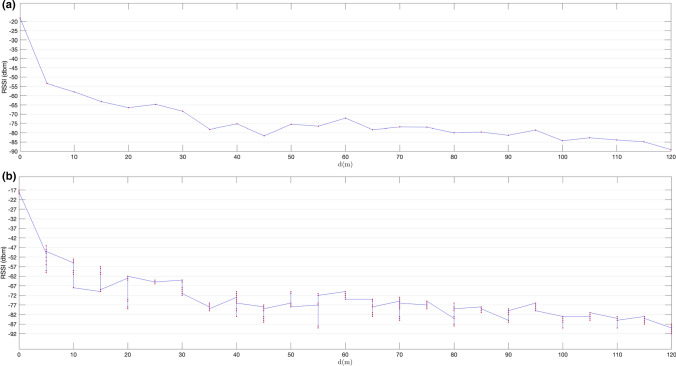
Mean RSSI (dBm) measured on received ADVREPORT (**a**); individual RSSI (dBm) measured on each ADVREPORT received (**b**); for a configured pTX = 0 dBm output power level, using primary extended advertisement over LE 1 M PHY

**Fig. 12:**
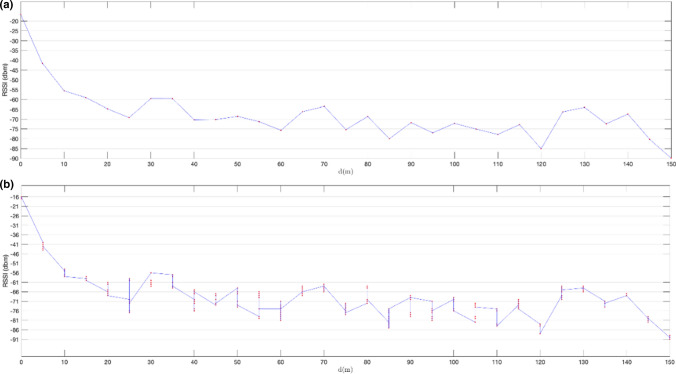
Mean RSSI (dBm) measured on received ADVREPORT (**a**); individual RSSI (dBm) measured on each ADVREPORT received (**b**); for a configured p TX = 8 dBm output power level, using primary extended advertisement over LE 1 M PHY

**Fig. 13:**
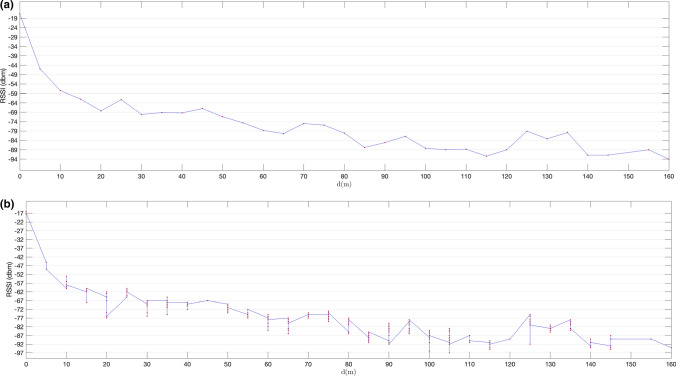
Mean RSSI (dBm) measured on received ADVREPORT (**a**); individual RSSI (dBm) measured on each ADVREPORT received (**b**); for a configured pTX = 0 dBm output power level, using primary extended advertisement over LE Coded PHY

**Fig. 14:**
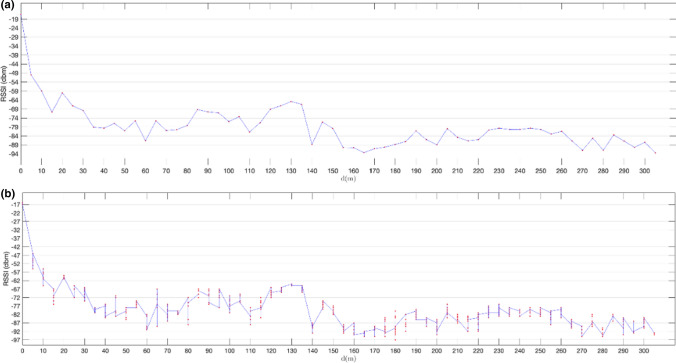
Mean RSSI (dBm) measured on received ADVREPORT (**a**); individual RSSI (dBm) measured on each ADVREPORT received (**b**); for a configured pTX = 8 dBm output power level, using primary extended advertisement over LE Coded PHY

In the first set of experiments, we used non-extended advertisements based on the system described in Sect. 5.2. The results of the tests detailed in Sect. 5.5 are shown in Figs. 11, 12, 13, 14. Figures 11a and 12a show mean RSSI values measured for advertisements received over LE 1 M PHY for a configured transmission power level of 0dBm and 8dBm respectively. Figures 11b and 12b show individual RSSI values measured for each advertising packet received at each distance. Figures 13 and 14 show the same results, but for primary extended advertisement over LE Coded PHY.

As expected, RSSI values are greater for higher transmission power levels. Longer distances are achieved by increasing power levels up to 8 dBm in the advertising nRF52840 DK board. With this transmission power, Figs. 12 and 14 show that, although signal strength continuously decreases with distance, RSSI values remain high enough to receive successful advertisements at considerable distances: 150 m when advertising over LE 1 M PHY and 310 m when advertising over LE Coded PHY. In Figs. 13 and 14, successful advertisements are shown at lower RSSI levels than in Figs. 11 and 12. This is caused by the characteristics of nRF52840 DK boards [23]. These boards have a sensibility of  − 103 dBm for communications conducted over LE Coded PHY, but in the case of communications over LE 1 M PHY, the sensibility of the receiver is -95 dBm.

The results obtained reveal longer ranges than those previously reported [1, 2]. These longer ranges are achieved by performing a connectionless advertisement-based communication, without the pairing process required in previous work. In connectionless communications there are no connection supervision timeouts forcing their termination when timers are exceeded. Thus, successful messages are eventually received even under unfavourable conditions. In the results shown in Fig. 11, a considerable decrease in the measured RSSI down to − 80 dBm is produced when the distance is equal to or greater than 45 m for LE 1 M PHY when using a transmission power level of 0 dBm. This coincides with the results reported in [1]. When the transmission power level is increased to 8 dBm, RSSI values remain above -80 dBm up to a distance of 85 m, as shown in Fig. 12. By conducting communications over LE Coded PHY, the distance up to which RSSI holds values above -80 dBm extends up to 80 m for a power level of 0 dBm and up to 140 m when using an output power of 8 dBm, as shown in Figs. 13 and 14 respectively.

**Fig. 15:**
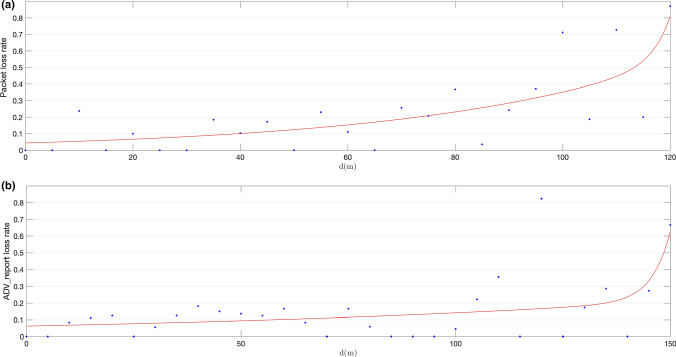
Calculated ADVREPORT loss rates, using primary extended advertisement on data channels over LE 1 M PHY for a configured pTX = 0 dBm output power level (**a**) and for a configured pTX = 8 dBm output power level (**b**)

**Fig. 16:**
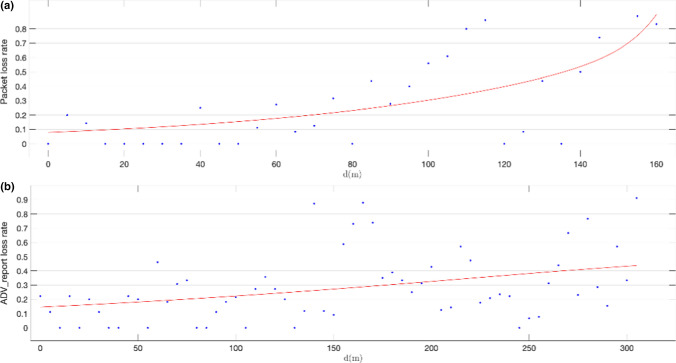
Calculated ADVREPORT loss rates, using primary extended advertisement on data channels over LE Coded PHY for a configured pTX = 0 dBm output power level (**a**) and for a configured pTX = 8 dBm output power level (**b**)

We also performed an estimation of packet loss rates, considering the configured advertising interval of 187.5 ms with a tolerance of 10 ms (the maximum random delay allowed by Bluetooth 5.x core specifications). The results are shown in Figs. 15 and 16, together with the corresponding two-term fit exponential model.

Figure 15 shows packet loss rates calculated for primary advertising over LE 1 M PHY using a transmission power level of 0 dBm (a) and 8 dBm (b) respectively. When the 0 dBm power level is set, packet loss rates remain below 0.35 up to a distance of 80 m between advertiser and observer. When the transmission power level is set to 8 dBm, this distance increases up to 120 m. Moreover, Fig. 16 shows packet loss rates obtained for primary advertising over LE Coded PHY using a transmission power level of 0 dBm (a) and 8 dBm (b) respectively. For communications over LE Coded PHY, packet loss rates remain under 0.35 up to 90 m for a transmission power of 0 dBm, and up to 140 m for 8 dBm. Nevertheless, there are isolated peaks that may have been caused by the effects on signal propagation of the buildings in the area where the experiments were conducted. In any case, the results demonstrate that packet loss rates at a given distance may be improved by increasing transmission power levels.

These results coincide partially with those reported by García et al. [20]. The results for LE Coded PHY are good for distances up to 300 m or even longer distances, but the results obtained with LE 1 M PHY are much worse. Nevertheless, they cannot be totally compared with our results because the authors have used an additional antenna attached to the boards used in the experiments and they have carried out their tests without interferences. Furthermore, we used fixed sized advertisements of 37 bytes, whereas the authors use other sizes. The authors even report a high variability in the results depending on the size of the messages.

### Throughput evaluation under static conditions

**Fig. 17:**
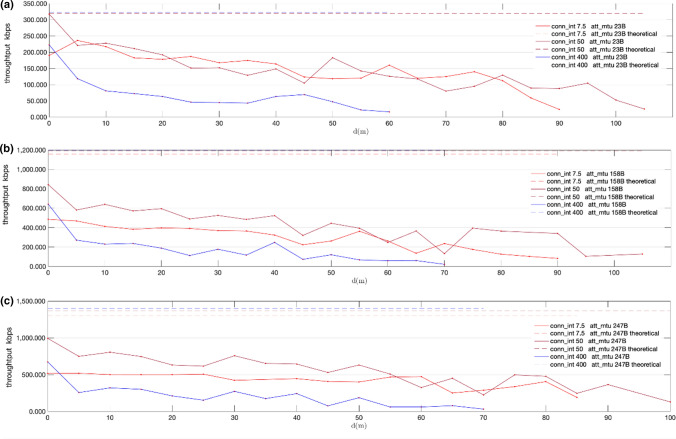
Measured throughput (kbps) for conn_interval = 7.5 ms, conn_interval = 50 ms and conn_interval = 400 ms, using given MTU sizes, over LE 2 M PHY: att_mtu = 23B (**a**); att_mtu = 158B (**b**); att_mtu = 247B (**c**)

**Fig. 18:**
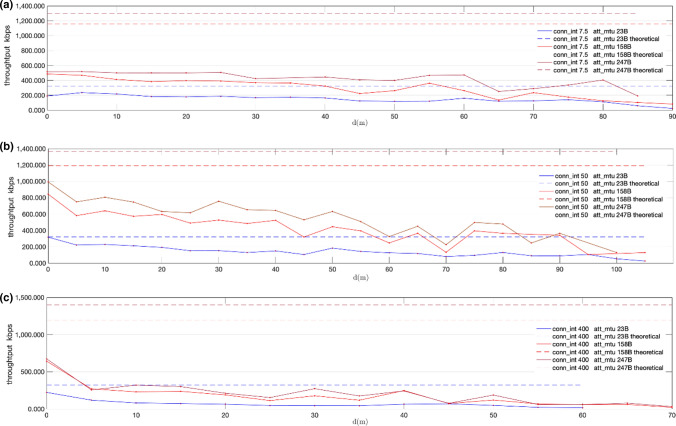
Measured throughput (kbps) for att_mtu = 23B, att_mtu = 158B and att_mtu = 247B, using given connection intervals, over LE 2 M PHY: conn_interval = 7.5 ms. (**a**); conn_interval = 50 ms. (**b**); conn_interval = 400 ms. (**c**)

**Fig. 19:**
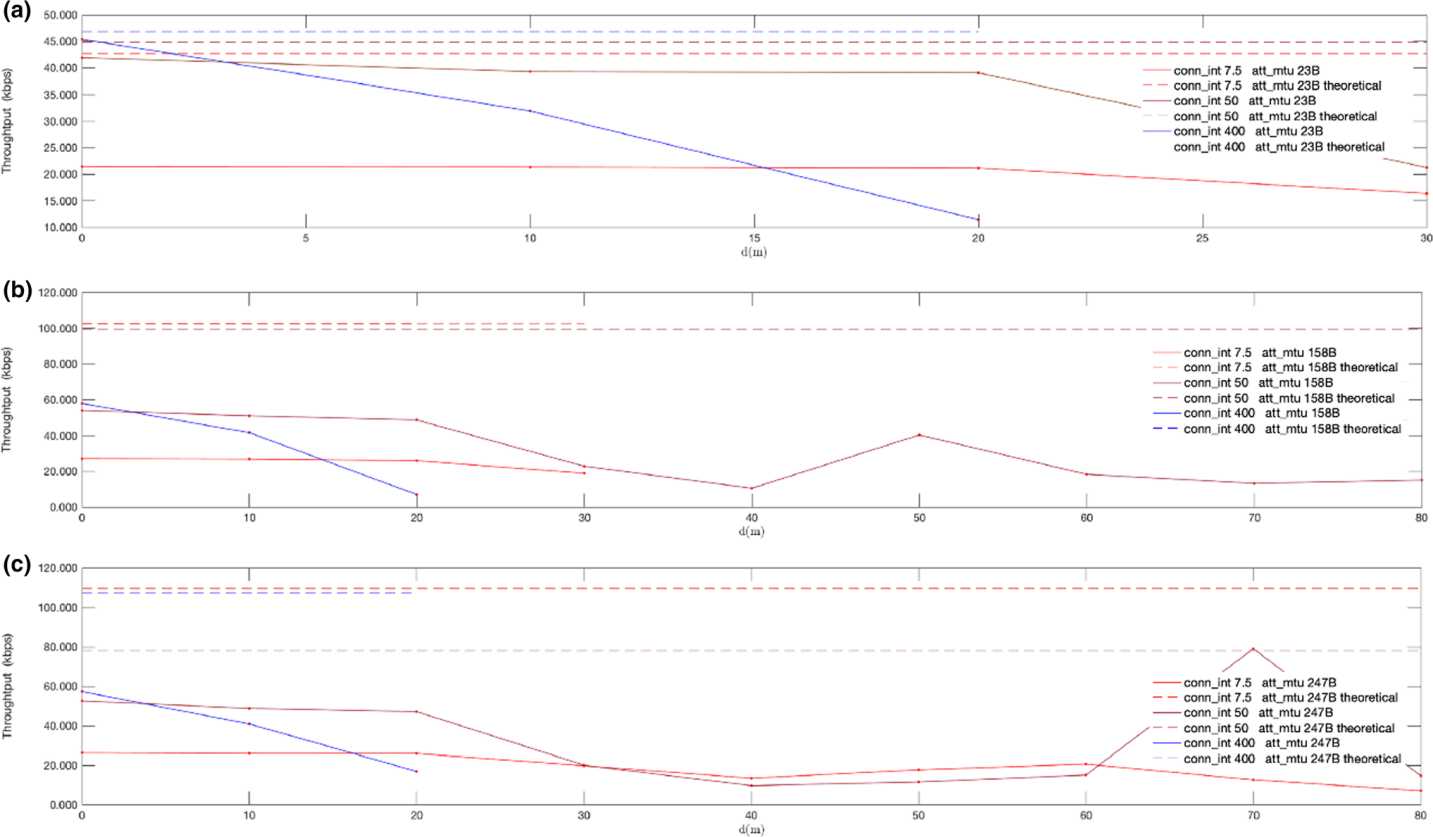
Measured throughput (kbps) for conn_interval = 7.5, ms, conn_interval = 50 ms and conn_interval = 400 ms, using given MTU sizes, over LE Coded PHY: att_mtu = 23B (**a**); att_mtu = 158B (**b**); att_mtu = 247B (**c**)

**Fig. 20:**
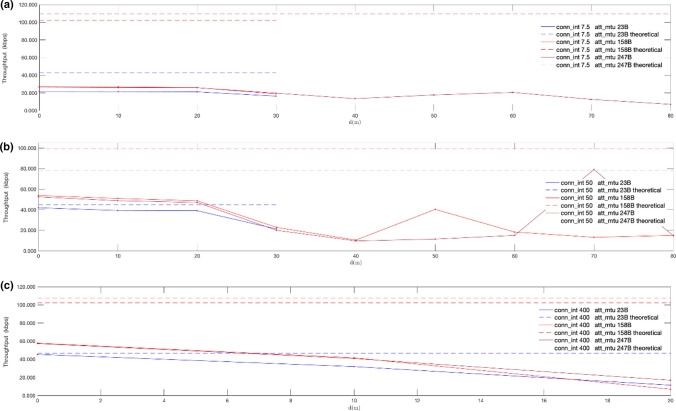
Measured throughput (kbps) for att_mtu = 23B, att_mtu = 158B and att_mtu = 247B, using given connection intervals over LE Coded PHY: conn_interval = 7.5 ms (**a**); conn_interval = 50 ms (**b**); conn_interval = 400 ms (**c**)

For the evaluation of the application throughput achieved with the new PHYs, we have used the connection-based system described in Sect. 5.3. The results of the tests detailed in Sect. 5.5 are shown in Figs. 17, 18, 19, 20. Note that each throughput line stops at a distance in which the time required to transmit a fixed amount of 256 KB causes a remarkable drop in throughput (below 8 kbps), due to the stop-and-wait flow control mechanism. The results are presented together with the theoretical throughputs calculated assuming DLE is enabled. They were calculated based on the time required to send a fixed number of bytes according to the packet format defined by core specifications [22].

Figures 17 and 19 show throughput values measured for fixed MTU values and different connection intervals for LE 2 M PHY and LE Coded PHY respectively. On the other hand, Figs. 18 and 20 show throughput values measured with fixed connection intervals and different MTU sizes for both LE 2 M PHY and LE Coded PHY.

As shown in the results, when the experiments are conducted over LE Coded PHY throughput drastically drops for distances equal to or larger than 20 m for several MTU and connection interval combinations. Thus, throughput tests were conducted for larger distances only using combinations of MTU sizes and connection intervals for which throughput still showed acceptable values. These combinations follow:

- 1.7.5 ms connection interval with MTU = 247 B
- 2.50 ms connection interval with MTU = 158 B
- 3.50 ms connection interval with MTU = 247 B

As expected, throughput decreases when distance increases due to the way transmission errors are handled as defined by core specifications. Moreover, packet rate fluctuations can also be explained given the retransmission strategy used. The stop-and-wait mechanism used degrades the effective throughput in the presence of bit errors, causing moderate throughputs to be achievable only for low connection interval values.

The transmission ranges achieved in the experiments conducted for both LE 2 M PHY and LE Coded PHY are shorter in comparison with those observed for the advertising based connectionless experiments shown in Sect. 6.1. The reason is that, in this case, if the 4-s GAP supervision timeout interval is exceeded, the communication between the central and peripheral boards is terminated, thus proving that connectionless communications permit longer distances.

Regarding the results obtained with LE 2 M PHY, Fig. 17 shows that the optimal value for connection intervals is 50 ms. Similarly, the results in Fig. 18 show that the optimal MTU is 247B. Nevertheless, the experiments conducted show negligible differences between 247 and 158B MTUs when the connection interval is increased up to 400 ms. This performance convergence is caused by the increase in bit error probabilities when using larger over-the-air packets. This reduces the impact of the time consumed sending extra overhead bits when using a 158B MTU, if compared with the wait time required for retransmissions in the connection interval following an error. Similar conclusions may be drawn when LE Coded PHY is used. Nevertheless, throughput values are generally lower than in the previous case, due to the usage of the S = 8 coding scheme.

Considering the results shown in Figs. 17, 18, 19, 20, an advertising based connectionless system using LE 2 M PHY with a connection interval of 50 ms and a MTU of 247B may achieve, at least, a throughput ranging from 992.5 kbps for a distance of 0 m to 128.9 kbps for a distance of 100 m. The expected throughput in a connectionless system for longer distances would be even better because stop-and-wait retransmissions will not occur.

### Performance evaluation under real driving conditions

**Fig. 21:**
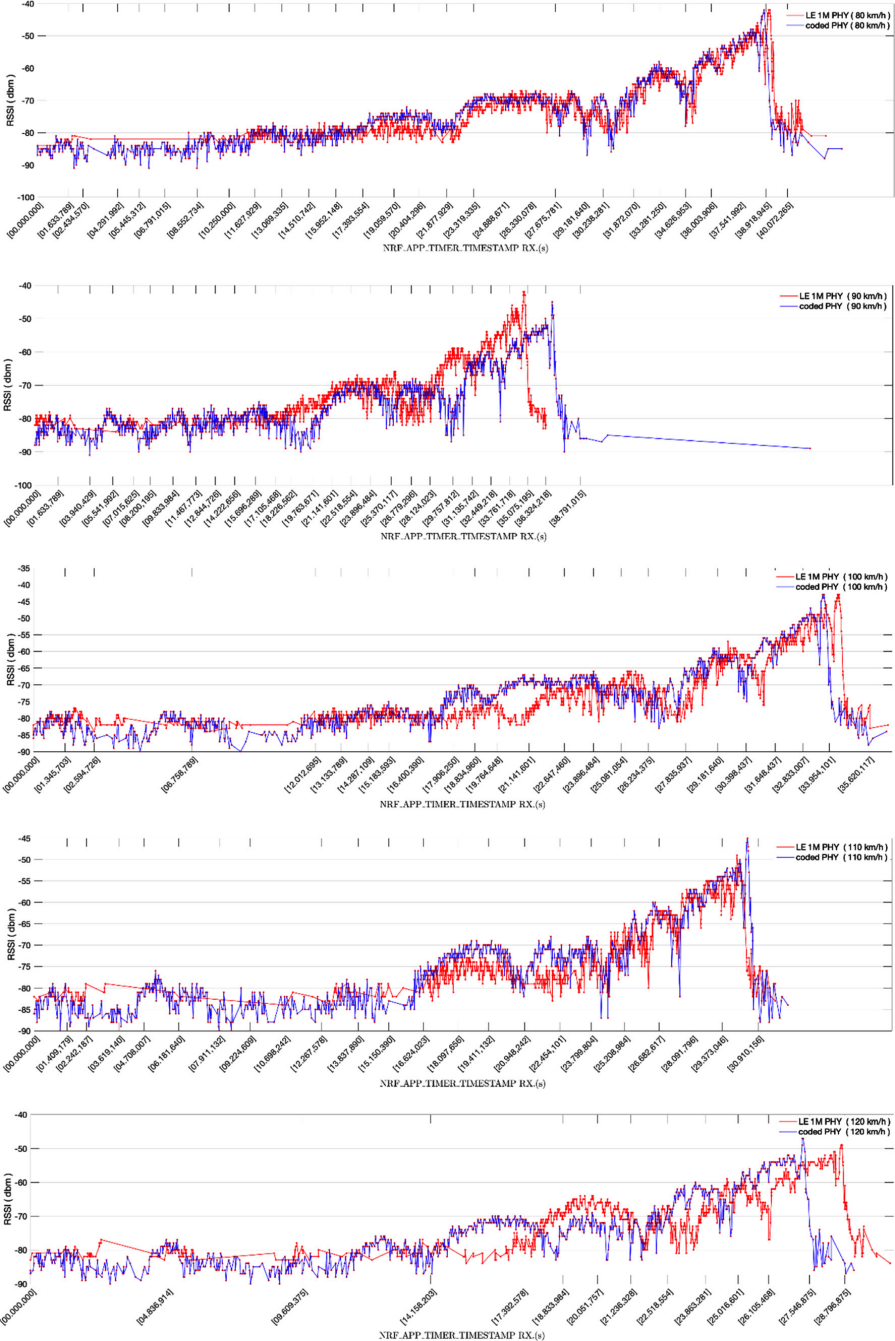
RSSI (dBm) measured advertising over LE 1 M PHY and LE Coded PHY for constant speeds ranging from 80 km/h (**a**) to 120 km/h (**e**)

**Fig. 22:**
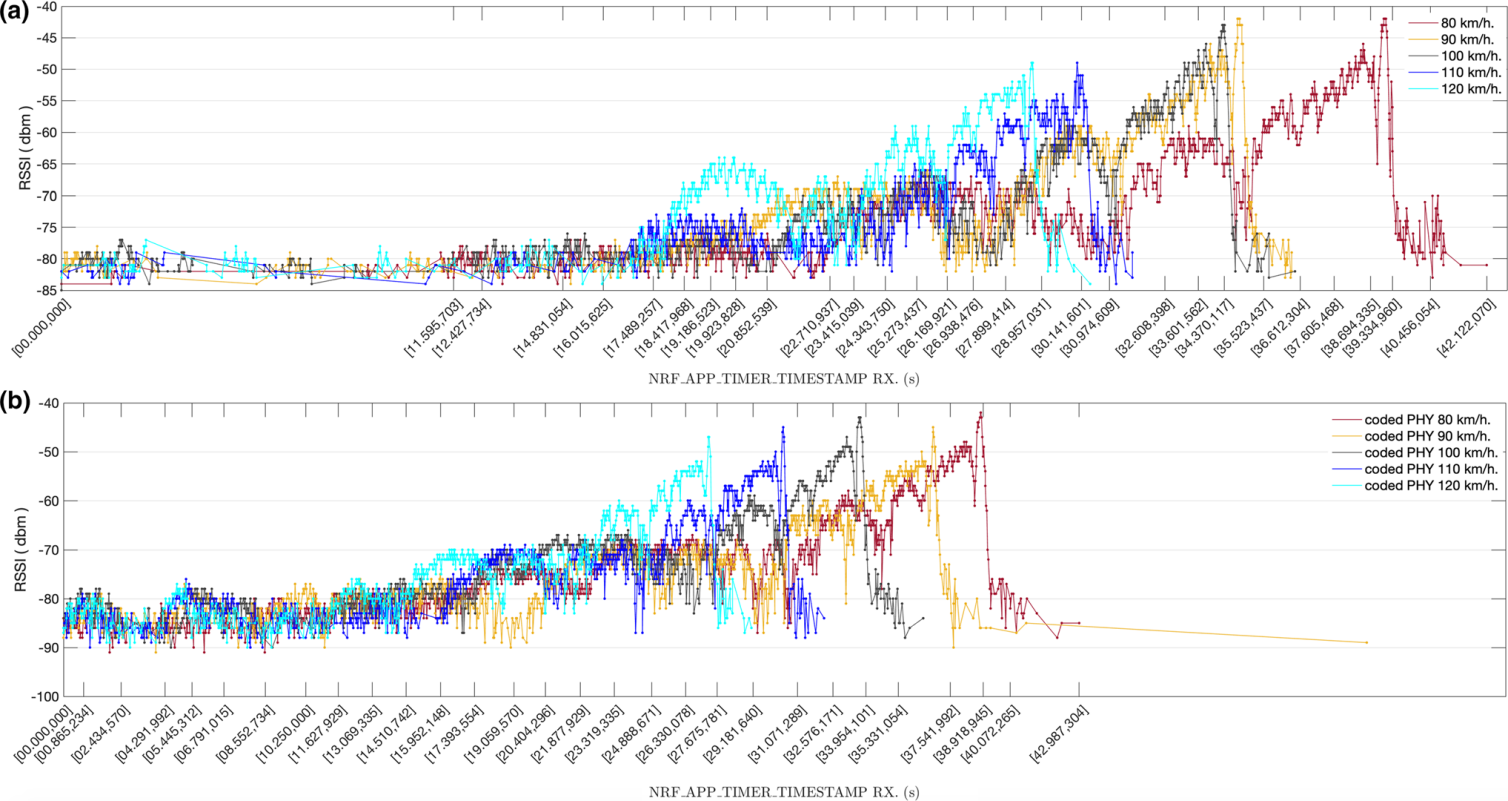
RSSI (dBm) measured advertising from the vehicle moving at constant speeds ranging from 80 km/h to 120 km/h, for both LE 1 M PHY(**a**) and LE Coded PHY(**b**)

In the last set of experiments, we also used non-extended advertisements based on the system described in Sect. 5.2 but with the advertiser board attached to a roof rack bar of a car and the observer board placed at a fixed position in a bridge over a major motorway, as described in Sect. 5.5. The results obtained are shown in Figs. 21 and 22. Figure 21 compares the RSSI values obtained using LE 1 M PHY with those obtained with LE Coded PHY, for constant speeds ranging from 80 km/h (a) to 120 km/h (e). Moreover, in Fig. 22 we compare the RSSI values obtained for each experiment speed, for both LE 1 M PHY (a) and LE Coded PHY (b). The results correspond to transmission windows that start when the observer board starts receiving advertisements. As expected, the average power measured decreases as relative speed increases (taking into account the reduction of the temporal transmission window and, thus, of the communication range).

The results show that for earlier advertisements the RSSI values vary between  − 80 dBm and  − 90 dBm. Furthermore, there is a higher density of advertisements when LE Coded PHY is used. This is caused by the enhanced sensibility of − 103dBm of nRf52840 DK boards vs the -95dBm for communications over LE 1 M PHY [23]. Considering the higher density of early packets received when longer distances separate both boards, we can assume that communications over LE Coded PHY may reveal even larger receiving time windows.

It was not possible to measure the distance between both boards accurately because nRF52840 DK boards do not include GPS positioning. Thus, this distance had to be estimated using the measured receiving time windows and the speed of the vehicle. Table 4 shows the estimated ranges corresponding to the results shown in Figs. 21 and 22. Note that the ranges achieved in the experiments are much larger than results presented in previous studies in which connection-oriented communications using a central/peripheral mode were used [2]. This proves that, in mobility scenarios, longer communication ranges may be obtained if an advertising connectionless based communication system is used.

**Table 4:** Estimation of achieved ranges achieved (m), for the observed transmission windows

Speed (km/h)	LE 1 M PHY	LE Coded PHY
120	1013 m	971 m
110	967 m	984 m
100	1013 m	1011 m
90	908 m	1379 m
80	936 m	955 m

The results also show that communications are feasible in highways in which a maximum speed of 120 km/h is permitted. The distance between a static device and an on-board device does not decrease rapidly enough to avoid the reception of a significant number of messages as stated in [1], in which a central/peripheral communication analysis is performed. Considering that the relative speed between two vehicles driving in the same direction is never greater than 120 km/h, not only V2I communications are feasible, but also a subset of V2V advertisement-based communications.

Further research is needed in order to analyse the communication ranges achievable by vehicles driving in opposite directions, thus determining data transfer capabilities using the proposed Bluetooth-based system for shorter transmission windows. In addition, the feasibility was proved for both LE 1 M PHY and LE Coded PHY with the S = 8 coding scheme, but additional experiments should be carried out to analyse the throughput obtained with all the different physical layers. Considering that LE Coded PHY was used with the S = 8 coding scheme, a higher density of successfully received advertisements may not be enough to compensate the redundancy introduced by the coding scheme, causing throughput to be cut down below the throughput achievable by advertising over uncoded LE 1 M PHY.

## Conclusions and future work

In this paper we analyse the potential of Bluetooth version 5 features for Inter-vehicular communications (IVC), providing results about its performance in terms of measured RSSI, packet loss rate and achieved range achieved under medium radio interference conditions. A prototype has been built with off-the-shelf nRF52840 DK boards and several experiments were carried out under real driving conditions in a major motorway with medium to dense traffic volume. These experiments were carried out under real interference conditions (hands-free devices, vehicles, smartphones and other Bluetooth devices) and with an interference setup designed for the experiments. Their design has allowed us to obtain realistic performance results and to check the scalability of the proposed solution.

Our proposal is based on a low-cost system based on an extended advertising mechanism designed for vehicular communications. The results show the convenience of a connectionless advertising-based approach for Bluetooth based VANET systems, instead of the more traditional central/peripheral connection-oriented approach. Furthermore, in terms of scalability this solution is better than connection-based solutions because there is an extremely low usage of the radio spectrum. The idea is having one broadcaster and multiple observers. The results obtained in the experiments reveal larger communication ranges, in comparison with those reported in previous work for BLE connection-based applications requiring a pairing mechanism.

During the experiments, we also analysed the performance of the proposed advertising-based system using both LE 1 M and LE Coded PHY layers under real driving conditions. The results demonstrate the feasibility of the envisioned system for vehicle to infrastructure communications in major motorway driving scenarios under medium to dense traffic conditions. Thus, it also proves the feasibility of these communications for vehicle-to-vehicle applications in city driving scenarios, with lower relative speed differences. Furthermore, we provide an analysis of the throughput achieved using different PHYs defined by Bluetooth 5.x core specifications, stablishing a reference minimum value for the throughput achievable by advertising-based systems.

As future work, we aim to conduct further research on determining the optimal PHY to be used in real driving scenarios. We would like to analyse the existing trade-off between the longer ranges achievable advertising with LE Coded PHY and the overhead introduced by the S = 2 or S = 8 coding schemes in contrast with the uncoded transmission scheme used when advertising over LE 1 M PHY or LE 2 M PHY. For such a purpose, we aim to adapt the implemented system to work with the latest releases of the *softdevice* of nRF52840 DK boards. These releases enable the boards to perform extended advertising data off-loading to secondary data channels. By upgrading the system to the new *softdevice* we aim to analyse the effect of increasing the amount of advertisable data on the achievable throughput. Also, we will be enabled to test the performance shown by the implemented system when advertising over LE Coded PHY using the S = 2 coding scheme.

Finally, we would like to analyse possible performance improvements or optimizations to the proposed system thanks to several characteristics included in versions 5.1 and 5.2 of the protocol, such as the Angle of Arrival (AoA) and Angle of Departure (AoD) [21]. This would enable devices to detect the direction of signals, enhancing positioning capabilities, and to estimate the distance between the boards. In addition, the randomized Advertising Channel Index feature may reduce interferences and collisions, enhancing the performance of the advertising-based system, particularly for large-scale Bluetooth mesh deployments.

## Data Availability

Data is available on demand.

## Code availability

Custom code.
